# Pernicious Anemia in an Adult with Trisomy 21

**DOI:** 10.1155/2023/2747756

**Published:** 2023-08-26

**Authors:** Kentaro Kamada, Osamu Kawano, Satoshi Yakuwa, Kentaro Wakasa, Kimiaki Uetake

**Affiliations:** ^1^Department of Pediatrics, Obihiro Kosei Hospital, Nishi 6-jo Minami 8-1, Obihiro, Hokkaido 080-0016, Japan; ^2^Okurayama-gakuin, 20-2 Miharashi-cho, Otaru, Hokkaido 047-0263, Japan; ^3^Department of Hematology, Obihiro Kosei Hospital, Nishi 6-jo Minami 8-1, Obihiro, Hokkaido 080-0016, Japan

## Abstract

Pernicious anemia is an autoimmune disease caused by the malabsorption of vitamin B12. It usually appears in the elderly. People with trisomy 21 are susceptible to autoimmune diseases. This susceptibility is thought to be due to altered expression of the *AIRE* gene, which is located in the 21q22.3 region. Although pernicious anemia is not common in people with trisomy 21, *AIRE* is pointed out as a susceptibility gene of pernicious anemia in a genome-wide association study. Here, we report a man with trisomy 21, who suffered from the pernicious anemia. When he was in his 30 s, he visited our hospital because of diarrhea and poor oral intake. He showed thrombocytopenic purpura-like features, and was diagnosed as pernicious anemia. After supplementation of vitamin B12, he recovered from the illness. The reason for his early onset may be because of trisomy 21. Altered expression of *AIRE* might trigger the disease.

## 1. Introduction

Pernicious anemia is an autoimmune disease caused by malabsorption of vitamin B12 manifesting megaloblastic anemia, neurological symptoms, and gastrointestinal symptoms. It usually appears in the elderly, most commonly between 60 and 80 years of age. Patients with trisomy 21 are susceptible to autoimmune diseases, such as Hashimoto's thyroiditis, Graves' disease, Type 1 diabetes, and celiac disease [1]. One reason for this susceptibility is thought to be due to the altered expression of the *AIRE* gene, which is located in the 21q22.3 region [2]. Here, we report a man with trisomy 21, who suffered from pernicious anemia in his 30 s.

## 2. Case Presentation

A man in his 30 s with trisomy 21, intellectual disability, autistic spectrum disorder, atrioventricular septal defect, and Eisenmenger syndrome visited our hospital because of diarrhea and poor oral intake. Although he was alert, he had hypotension (83/52 mmHg) and a low-percutaneous oxygen saturation of 70% with a 10 L/min O_2_ face mask; usually his oxygen saturation is between 85% and 90% in room air. Physical examination revealed icteric skin and conjunctiva, a dehydrated mouth, and clubbed fingers. He had petechiae on the chest, but no spontaneous bleeding was found. A Levine III/VI holosystolic murmur was heard on the third left sternal border.

He admitted to our hospital, and the initial evaluation demonstrated megaloblastic anemia (hemoglobin, 9.8 g/dL; mean corpuscular volume, 129.4 fL); normal reticulocyte count (90.2 × 10^9^/L); thrombocytopenia (52 × 10^9^ /L); unconjugated hyperbilirubinemia (total bilirubin, 4.3 mg/dL; direct bilirubin, 0.3 mg/dL); elevated serum lactate dehydrogenase (1,755 U/L); and decreased serum haptoglobin (<100 mg/L). Before the present episode, he had polycythemia due to chronic cyanosis caused by uncorrected atrioventricular septal defect and Eisenmenger syndrome. The latest blood test was 8 months prior to the admission, and hemoglobin was 20.1 g/dL at that time. Therefore, 9.8 g/dL of hemoglobin represented the acute anemia. Although hemolysis was indicated, his reticulocyte count was 90,200 /*μ*L and was not increased. His blood urea nitrogen was 37.1 mg/dL and serum creatinine was 1.29 mg/dL, showing acute kidney injury. We checked hemorrhage as a cause of anemia since he had thrombocytopenia, but no hemorrhage was detected.

Although we initially suspected thrombotic thrombocytopenic purpura (TTP), we diagnosed his illness as pernicious anemia for the following reasons. First, the acute kidney injury was resolved by intravenous rehydration. Second, decreased serum vitamin B12 (107 pg/mL), and elevated serum total homocysteine (55.6 mmol/mL) were revealed. Third, the antiparietal cell antibody and intrinsic factor antibody were both positive. Fourth, his ADAMTS13 activity was 30%, whereas TTP is diagnosed when the activity is lower than 5%.

**Figure 1:**
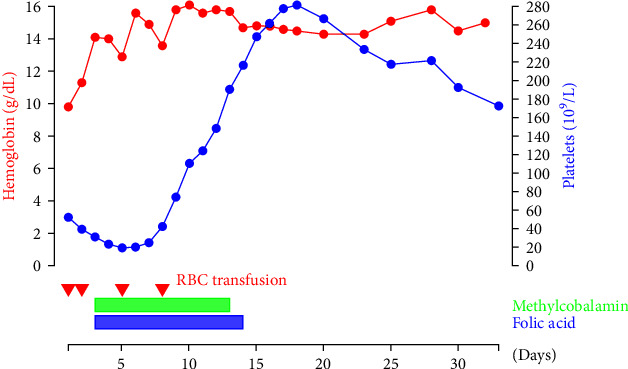
Timeline summary showing improvements in the patient's anemia and thrombocytosis demonstrated by his hemoglobin level and platelet counts. Red blood cell was transfused on the 1st, 2nd, 5th, and 8th hospital day. Methylcobalamin and folic acid were supplemented intramuscularly from the 3rd hospital day until his blood concentration increased above the upper normal range. His platelet counts started to increase from the 6th hospital day.

Soon after reaching the diagnosis, methylcobalamin was supplemented intramuscularly. Since his optimal hemoglobin level was calculated to be 19 g/dL because his oxygen saturation was 85% in normal time, red blood cells were transfused (Figure 1). In addition to methylcobalamin, folic acid was also supplemented because his serum folate level was decreased (1.2 ng/mL). After decreasing to 10 × 10^9^/L, the platelet count started to increase from the 5th hospital day. The anemia resolved gradually and the last red blood cell transfusion was on the 8th hospital day. On the 14th hospital day, supplementation of methylcobalamin and folic acid ceased because the patient's serum vitamin B12 and folate levels exceeded the upper normal limit. In the course of recovery, exacerbation of preexisting chronic pericarditis and decreased swallowing function emerged. After coping with those problem, he was discharged home on the 33rd hospital day. Since then, the anemia or thrombocytopenia did not relapse with intramuscular methylcobalamin supplementation every 3 months.

## 3. Discussion

Pernicious anemia is an autoimmune disease characterized by the impaired absorption of vitamin B12 (methylcobalamin) and caused by the absence of gastric intrinsic factors. It results in megaloblastic anemia, glossitis, atrophic gastritis, peripheral neuropathy, and other neurological deficits. Sometimes pernicious anemia presents with a TTP-like manifestation. It is characterized by anemia and thrombocytopenia with elevated serum indirect bilirubin, lactate dehydrogenase, and decreased haptoglobin. It is hypothesized that an ineffective hematopoiesis results in these manifestations, and not the thrombotic mechanism seen in the thrombotic microangiopathy such as TTP [3].

Since pernicious anemia is usually seen in elder people in their 60s and 70s, the present case may have a factor to promote the onset of the disease. Trisomy 21 is frequently associated with autoimmune diseases, such as celiac disease, Type 1 diabetes, and thyroiditis. We found a single article mentioning the association between trisomy 21 and pernicious anemia [4]. However, we could not find any case report or specific description of the coexistence of trisomy 21 and pernicious anemia. There is a gene called *autoimmune regulator* (*AIRE*) on 21q22.3. *AIRE* is expressed in the thymus and it encodes a protein that regulates central immunological tolerance [2]. *AIRE* promotes the negative selection of T lymphocytes by promoting the transcription of tissue-specific antigens, therefore, the dysfunction of *AIRE* may result in autoimmune diseases. For example, variants of *AIRE* cause autoimmune polyendocrine syndrome type-1 (APS-1) in an autosomal recessive manner. APS-1 is characterized by the chronic mucocutaneous candidiasis, hypoparathyroidism, and autoimmune adrenal insufficiency. APS-1 also manifests pernicious anemia in some patients. During a genome-wide association study meta-analysis, *AIRE* was pointed out as a susceptibility gene of pernicious anemia [5]. Although there are three alleles of *AIRE* in people with trisomy 21, its expression is rather decreased in the thymus compared to the normal controls [6, 7]. This may result in insufficient central immunological tolerance and early onset pernicious anemia. As the life expectancy of people with 21 trisomy is increasing over the years, we may encounter pernicious anemia in people with trisomy 21 more frequently than before. We should keep in mind that pernicious anemia may occur in relatively young adults with trisomy 21.

## Data Availability

The datasets generated during and/or analyzed during the current study are available from the corresponding author on reasonable request.
